# Impact of Clinical Decision Support Systems on Medical Students’ Case-Solving Performance: Comparison Study with a Focus Group

**DOI:** 10.2196/55709

**Published:** 2025-03-18

**Authors:** Marco Montagna, Filippo Chiabrando, Rebecca De Lorenzo, Patrizia Rovere Querini, Medical Students, Mariam Datukishvili

**Affiliations:** 1 School of Medicine, Vita-Salute San Raffaele University, Via Olgettina 58, Milan, 20132, Italy; 2 Unit of Medical Specialties and Healthcare Continuity, IRCCS San Raffaele Scientific Institute, Milan, Italy

**Keywords:** chatGPT, chatbot, machine learning, ML, artificial intelligence, AI, algorithm, predictive model, predictive analytics, predictive system, practical model, deep learning, large language models, LLMs, medical education, medical teaching, teaching environment, clinical decision support systems, CDSS, decision support, decision support tool, clinical decision-making, innovative teaching

## Abstract

**Background:**

Health care practitioners use clinical decision support systems (CDSS) as an aid in the crucial task of clinical reasoning and decision-making. Traditional CDSS are online repositories (ORs) and clinical practice guidelines (CPG). Recently, large language models (LLMs) such as ChatGPT have emerged as potential alternatives. They have proven to be powerful, innovative tools, yet they are not devoid of worrisome risks.

**Objective:**

This study aims to explore how medical students perform in an evaluated clinical case through the use of different CDSS tools.

**Methods:**

The authors randomly divided medical students into 3 groups, CPG, n=6 (38%); OR, n=5 (31%); and ChatGPT, n=5 (31%); and assigned each group a different type of CDSS for guidance in answering prespecified questions, assessing how students’ speed and ability at resolving the same clinical case varied accordingly. External reviewers evaluated all answers based on accuracy and completeness metrics (score: 1‐5). The authors analyzed and categorized group scores according to the skill investigated: differential diagnosis, diagnostic workup, and clinical decision-making.

**Results:**

Answering time showed a trend for the ChatGPT group to be the fastest. The mean scores for completeness were as follows: CPG 4.0, OR 3.7, and ChatGPT 3.8 (*P*=.49). The mean scores for accuracy were as follows: CPG 4.0, OR 3.3, and ChatGPT 3.7 (*P*=.02). Aggregating scores according to the 3 students’ skill domains, trends in differences among the groups emerge more clearly, with the CPG group that performed best in nearly all domains and maintained almost perfect alignment between its completeness and accuracy.

**Conclusions:**

This hands-on session provided valuable insights into the potential perks and associated pitfalls of LLMs in medical education and practice. It suggested the critical need to include teachings in medical degree courses on how to properly take advantage of LLMs, as the potential for misuse is evident and real.

## Introduction

Clinical reasoning and decision-making are at the core of the medical workflow. If they are accurate and grounded on solid and updated evidence, they help ensure the best health outcomes for patients. Clinical decision support systems (CDSS) have been implemented to aid practitioners in this duty [1-3]. Clinical practice guidelines (CPG) serve as the prototype for CDSS. They are published and updated at varying frequencies by scientific societies and policy makers, covering virtually every medical field or disorder. Over time, the number and complexity of CPG have increased, resulting in more detailed and robust recommendations. However, this has also led to reduced immediacy and ease of access and comprehension for medical professionals. Additionally, there may be multiple CPG for a single pathological condition, sometimes with conflicting recommendations. As a potential solution, emerging technologies on the internet have given rise to new CDSS options known as online repositories (ORs). These repositories, like encyclopedias, consolidate and synthesize knowledge related to various medical disorders. They draw from current practices, available CPG, and the latest published evidence, making this information easily accessible to physicians. Typically provided by publishing groups, ORs often require subscription-based access. Two of the most popular options are “UpToDate” (by Wolters Kluwer [4]) and “BMJ Best Practice” (by BMJ Publishing Group [5]), both available as websites and mobile apps. The recent introduction of large language models (LLMs) for public use has generated both excitement and debate. Their adoption has rapidly grown across various human activities [6]. Many foresee the immense potential benefits of applying such technology to medical practice, while others harbor concerns about the dangers it might pose if left unregulated and misaligned [7-12].

Without a doubt, LLMs like ChatGPT represent a new generation of CDSS with unparalleled assistance capabilities. They can engage in active interactions with users and directly interpret medical information, extending far beyond simple guideline consultation. They can suggest possible diagnostic workups (DWs) or treatment algorithms [6]. In such cases, physicians would no longer need to navigate extensive datasets of clinical information, distill practical advice from lengthy text pages, or grapple with uncertainty about consulting the correct or sufficient sources. On the flip side, it is evident that LLMs also carry the potential for misuse, which could lead to significant harm to patients [7-9,11,13]. There’s a risk of guiding clinicians down erroneous thought processes, potentially resulting in wasted time and the unintentional complication of cases. When the alternatives being evaluated are either incorrect or become excessively numerous, the complexity of a case may inevitably worsen. As a result, there is legitimate concern regarding how the indiscriminate use of LLMs might inadvertently drive-up health care costs. This underscores the importance of integrating LLMs into clinical decision-making (CDM) processes with caution and judiciousness.

However, although the adoption of innovative CDSS tools is steadily rising, the lack of dedicated training in their proper utilization undermines their full potential as valuable aids [9,10].

The aim of the present study was to investigate how senior medical students employ CDSS in the resolution of a clinical case with the ultimate intention of designing specific educational programs. Specifically, we conducted a hands-on session to compare CPG, ORs, and an LLM (ChatGPT) in terms of speed and accuracy of the clinical decisions proposed after consultancy with the CDSS.

## Methods

### Study Design

The present is a report of a hands-on practical session taking place during the Course of Internal Medicine at our university. The subject of the analysis was the quality of students’ answers to a number of open-ended questions related to clinical reasoning and problem-solving, as a proxy for their capacity to employ different CDSS. A fictional clinical case was designed by the authors to control for complexity. Additionally, ChatGPT (version 3.5) generative capabilities were used to fabricate vital parameters, physical examination, and laboratory results for the fictional patient. ChatGPT was asked to include confounding factors in the answers provided. The authors revised generated elements to make sure they met the study requirements. The complete clinical case, open-ended questions and conversation with ChatGPT are available in Multimedia Appendix 1 and Multimedia Appendix 2.

### Participants, Recruitment Strategies, and Sampling Method

Students attending the last lesson in the academic year 2022/23 Course of Internal Medicine of the International MD Program degree at Vita-Salute San Raffaele University, Milan (IT), were all included in the study, with convenience sampling. No exclusion criteria were applied.

### Experimental Groups, Randomization, and Blinding

In total, 3 groups with comparable numbers of students were defined at the beginning of the lesson (Multimedia Appendix 3). Starting from the first seating rows, students were randomly assigned a number from 1 to 3 and consequently formed the 3 groups. Each group nominated a delegate who randomly picked an envelope containing the indication of the type of CDSS to be used by his or her group, either (i) CPG, (ii) OR, or (iii) ChatGPT. For each group, only the delegate was allowed web-based access to the CDSS. Group assignments were open label. The group assigned to CPG was allowed to use the internet to search for and consult CPG deemed useful to solve the case. The group assigned to OR was allowed to use the internet to access UpToDate and look for articles and algorithms or tables deemed useful to solve the case. The group assigned to ChatGPT was allowed to log into the LLM and use it to ask and gather information deemed useful to solve the case.

The delegate was also in charge of sending his or her group’s clinical decision to the researchers via a mobile phone SMS text message. An inspector of the research staff was assigned to each group to guarantee that only the assigned CDSS was used. The questions were shown along with the presentation of the clinical case in a Microsoft PowerPoint (Microsoft Corporation) slideshow. For each question, a countdown timer was shown on the projector screen, and the start time was recorded by the researchers. Time required by each group for each answer was calculated by subtracting the start time from the mobile message arrival time.

### Outcomes/Assessment

In total, 1 junior resident in internal medicine, 1 senior resident in internal medicine, and 2 internal medicine junior consultants were asked to perform blind external assessment of the answers. They were provided with a form containing the same clinical case and questions shown to the students together with the answers given by each group, with no details on CDSS used. Answers were graded from 1 to 5 in terms, respectively, of completeness and accuracy. Completeness was described as: “Is the answer complete or does it miss anything?”. Accuracy was described as: “Is the answer precise and adherent to clinical practice, or too vague, too wide, too superficial?”

### Sample Size

Sample size was not defined a priori. Sample size was determined by the number of students that attended the lesson on that day.

### Statistical Analysis

Scores are reported as mean. For further analysis, the 8 questions were grouped into 3 domains according to the students’ skill investigated: (1) differential diagnosis (DD, Q 1-5-6), (2) DW (Q 2‐7), and (3) CDM (Q 3-4-8). The Kruskal-Wallis test was performed to establish whether there was a significant difference among the 3 groups in the times and scores overall and in each student skill domain. Microsoft Excel (Microsoft Corporation) and GraphPad Prism (version 9.0; GraphPad Software) were used as software tools for the analysis.

### Ethical Considerations

This study does not require ethical approval as this is a report of data collected for monitoring and reporting purposes of innovative teaching activities taking place at Vita-Salute San Raffaele University, according to the Self-assessment-Evaluation-Accreditation system (AVA) of ANVUR, to which our institution is subject [14]. The data were generated during a hands-on session taking place during a usual lesson and in a teaching, non-experimental environment, with no risks for the participants. Data represent the output of each student group; they therefore collect aggregated information, with no individual identity linked to them. No confidential data were collected. No compensation was provided to participants, and they were able to opt out at any time during the lesson. AVA aims to improve the quality of teaching and research carried out in the Italian universities through the application of a quality assurance model based on internal procedures for planning, management, self-assessment, and improvement of training and scientific activities and on an external verification carried out in a clear and transparent manner. The requirements of the new AVA3 model underline the importance for the universities to promote, support, and monitor the participation of teachers in training and teaching refresher initiatives in the various disciplines, including those relating to the use of innovative teaching methodologies, also through the use of online tools and the provision of multimedia teaching materials [14]. The presented data were collected in this context, and, accordingly, no ethics approval was applied for (Page 3, Art.5, Clause 2 of [15]).

## Results

A total of 16 students were included: 6 allocated to the CPG group (*F*=5, 83%), 5 to the OR group (*F*=2, 40%), and 5 to the ChatGPT group (*F*=3, 60%).

During the presentation of the clinical case, all 3 groups were presented with questions, and students were required to provide their responses as quickly as possible, within predefined time limits. The time taken to answer each question was recorded for all groups. Except for one response, all answers were given within the allocated time (see Table 1). Of the 49 total allocated minutes, the CPG group took 41 minutes to complete the clinical case, the OR group 45 minutes, and the ChatGPT group 38 minutes. The total time taken to answer, expressed as a percentage of the allocated time, was not significantly different among groups (*P*=.69).

**Table 1.** Time (min) required for answers and mean score received at the external assessment in terms of completeness and accuracy for each answer given by each group. Overall time for completion and median score across all answers for each group are also reported. Allotted time was exceeded only in Q4 by OR group.

	Clinical practice guidelines			Online repositories			ChatGPT		
	Time (min)	Quality		Time (min)	Quality		Time (min)	Quality	
		Completeness, mean (SD)	Accuracy, mean (SD)		Completeness, mean (SD)	Accuracy, mean (SD)		Completeness, mean (SD)	Accuracy, mean (SD)
Q1. Rank the possible differential diagnoses in terms of probability.8 min^a^	8	4.0 (0.8)	3.8 (1.3)	8	3.5 (0.6)	3.0 (0.8)	8	4.0 (0.8)	3.8 (0.5)
Q2. Based on the previous list, which diagnostic workup would you set up?8 min^b^	7	4.0 (0.8)	3.8 (0.5)	7	4.8 (0.8)	2.5 (1.0)	6	3.8 (0.5)	3.8 (1.3)
Q3. Which values are altered?5 min^c^	3	4.0 (0.8)	4.3 (1.0)	5	3.8 (1.0)	4.0 (1.2)	2	4.0 (0.8)	2.3(1.3)
Q4. Which treatment do you start?5 min^c^	5	4.3 (1.5)	3.8 (1.5)	6	4.0 (0.8)	4.3 (1.0)	4	4.0 (0.8)	4.3 (1.0)
Q5. Which are the possible causes of hypercalcemia?8 min^a^	5	3.8 (1.0)	3.8 (1.0)	4	3.8 (0.5)	3.8 (1.0)	6	3.0 (0)	3.3 (1.3)
Q6. Can you narrow down the previous list based on these findings?5 min^a^	3	4.0 (1.4)	4.3 (1.5)	5	3.0 (0.8)	2.0 (0.8)	5	4.3 (1.0)	4.0 (0.8)
Q7. Which are the primary diagnostic tests that you order?5 min^b^	5	4.3 (1.0)	4.5 (1.0)	5	3.8 (1.5)	3.4 (0.7)	2	4.0 (0.8)	4.3 (0.9)
Q8. Which therapeutic choice do you offer to the patient?5 min^c^	5	3.8 (0.5)	4.3 (1.0)	5	3.5 (1.3)	3.4 (1.3)	5	3.8 (1.0)	4.0 (1.1)
TOT/mean^a^	41	4.0 (0.2)	4.0^d^ (0.3)	45	3.8 (0.5)	3.3^d^ (0.8)	38	3.8 (0.4)	3.7^d^ (0.7)

a Differential diagnosis

b Diagnostic workup

c Clinical decision-making

d *P*=.02.

**Figure 1.**
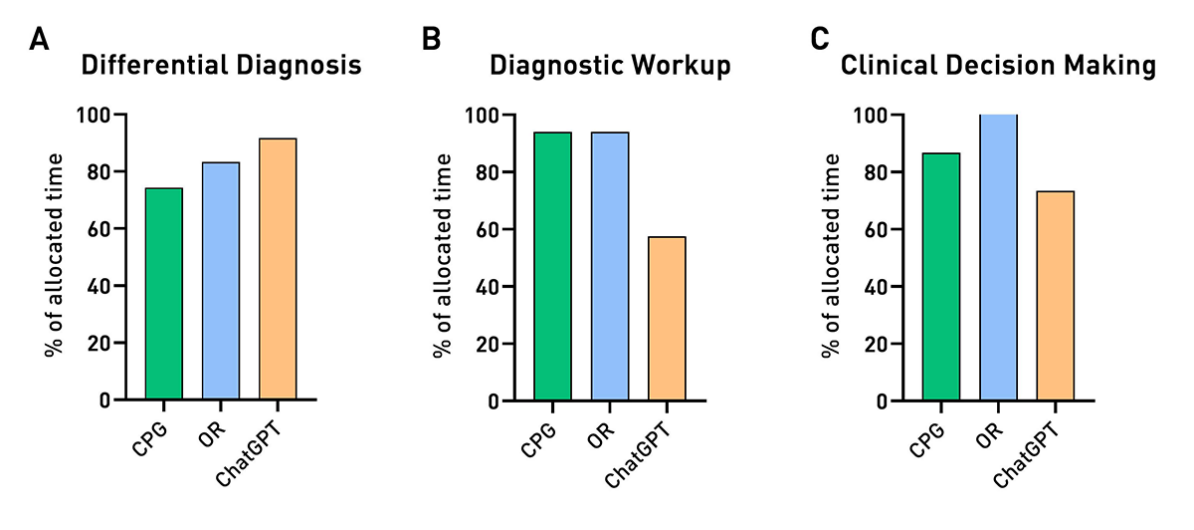
Sum of the time taken by the 3 groups of students to answer questions in the 3 domains. Results are shown as the percentage of the total allocated time for that domain. CPG: clinical practice guidelines; OR: online repositories.

The questions were then categorized into 3 major domains: DD (Q 1-5-6), DW (Q 2‐7), and CDM (Q 3-4-8). The time taken to answer, as a percentage of the allocated time, by each group of students according to the provided domains is shown in Figure 1(A, B and C). While no statistically significant differences were observed, it is worth noting that the ChatGPT group tended to respond more quickly to questions related to DW and CDM.

**Figure 2.**
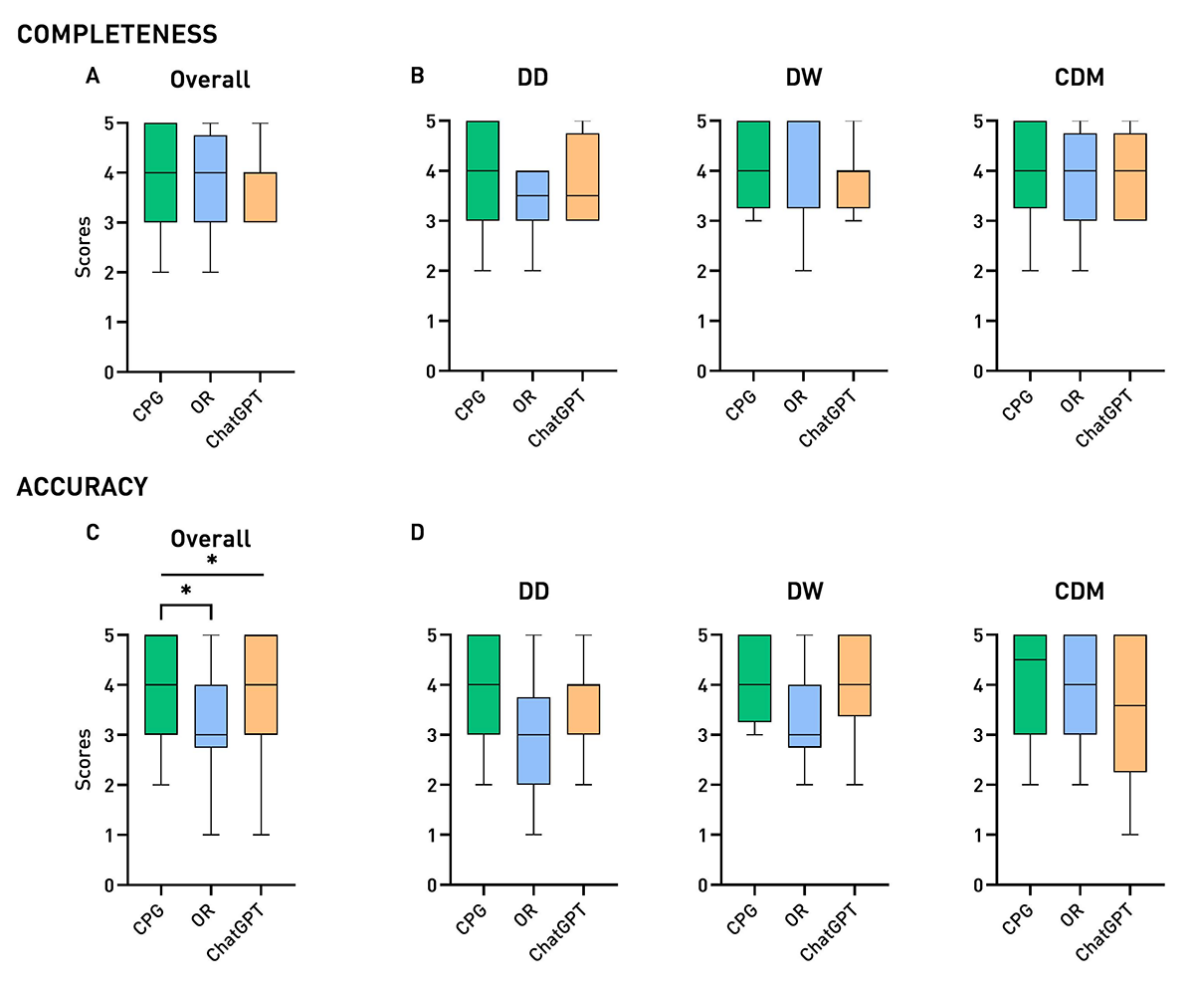
Box plots of scores obtained by the 3 groups of students for (A) overall completeness, (C) overall accuracy, (B) completeness in the 3 domains, (D) accuracy in the 3 domains. DD: differential diagnosis; DW: diagnostic workup; CDM: clinical decision-making (*P<.05).

Answers were assessed for completeness, which considered the depth of information provided, and for accuracy, which assessed adherence to clinical practice versus an excess or superficiality of information. These evaluations were conducted by 4 external reviewers who were blinded to the group assignment. Scores are reported in Table 1 and Figure 2.

Overall, the CPG group performed best, reaching the highest mean scores for completeness (4.0) and accuracy (4.0) (Figure 2A and C). The ChatGPT group comes in second place, with equal completeness (3.8 vs 3.8) compared to the OR group but higher accuracy (3.7 vs 3.3). The Kruskal-Wallis test showed non-significant difference among groups for overall completeness (*P*=.49), and a significant difference in overall accuracy between the 3 groups (*P*=.02), particularly—at post hoc analysis—between the CPG and the OR group (*P*=.02).

Aggregating scores according to the 3 students’ skill domains, trends in differences among the groups emerge more clearly (Figure 2B and C). When it comes to generating differential diagnoses, the CPG group was the most complete (3.9) and accurate (3.9) among the 3, whereas the OR group has the worst scores for both completeness and accuracy categories, with mean scores of 3.4 and 2.9, respectively, among the lowest registered. On the contrary, whenever students were asked to provide a DW for the patient, the OR group appeared as the most complete (4.3), although the least accurate (3.0), considering the source of the competition. In this domain, the other 2 groups come quite close (4.1 and 4.1 for CPG; 3.9 and 4.0 for ChatGPT) and maintain coherent scores in between their own completeness and accuracy. Lastly, when knowledge had to be applied to drive clinical decisions, as tested in CDM questions, the 3 groups obtained similar scores in terms of completeness, while the CPG group succeeded as the most accurate (4.1), followed by the OR group (3.9) and the ChatGPT group (3.5).

Of note, it appears evident how the CPG group performed the best in nearly all domains and maintained almost perfect alignment between its completeness and accuracy. The ChatGPT group maintained an overall mediocre yet stable performance, without ever achieving the best scores. On the other hand, the OR group showed mixed features, with notable peaks of performance–with scores equal to or higher than the ChatGPT contender–but some other dramatic drops in answer quality in terms of accuracy in more than one skill domain.

## Discussion

In recent years, the availability of tools to support clinicians in the diagnostic and therapeutic processes has grown considerably. Although the use of CDSS is widespread, individuals often use them without specific education and pay little attention to their inherent limitations, especially in the case of their newest potential counterparts, such as ChatGPT [1].

To assess how final-year medical school students make use of the available CDSS and to begin considering an instructional approach for the use of such tools, we designed the experiment outlined in this study. Observing the students’ interaction with the assigned CDSS during the resolution of a clinical case and analyzing their answers, we recorded specific criticalities regarding the use of each CDSS.

In terms of rapidity of use, ChatGPT seems to represent a significant breakthrough in the world of CDSS. If traditional encyclopedic or textbook-like written resources call the reader to go through the entire material to properly understand a topic and retrieve correct clinical answers or guidance, an instantaneous chat environment allows for both a quick overview of disciplines and—at the same time—deep vertical dives into specific details. Students using ChatGPT arrived at the required answer almost always faster than their colleagues aided by CPG or OR, especially in questions regarding DW and CDM. It seems that the role of ChatGPT in the chat dialogue more closely resembles the attitude of a human counterpart (for example, a senior teaching physician asked for guidance), whose responses are fast, direct, and usually finely targeted. Such answers follow the students’ line of reasoning and indirectly encourage them to choose a unique path of solution out of many possible scenarios. This dynamic emerges as brilliantly effective whenever the students embark on the correct clinical thought process but can lead to disastrous consequences whenever students feed cognitive biases or overt errors into their chat conversation. On the contrary, CPG and OR offer vast amounts of information, such as long lists of items and in-depth descriptions, whose digestion is neither easy nor fast. Therefore, whenever consulted correctly and for enough time, CPG and OR—especially the former, our results seem to suggest—generally help students give answers of higher quality, both in terms of completeness and accuracy. No real-time interaction is present; therefore, they appear almost immune to reader-introduced bias and misinterpretation.

In the modern era, velocity is a precious commodity, especially in the fast-paced clinical context, where less and less time is available for extensive reference consultation. This might influence current and future generations of medical school students to prefer chatbot-based guidance over preformed texts as routine help throughout their study [1,8].

As said, in terms of highest answer quality, there seems to be no rival to CPG. Old-fashioned guidelines might be slower to consult but grant far greater quality information to students, helping them to be complete and accurate in key tasks, such as generating differentials and deploying clinical decisions. Possibly, CPG might also enhance the student’s comprehension of the analyzed topic, given the broader context and deeper description always provided. Nonetheless, in a continuously evolving context of increasing number and complexity of CPG, it would be presumptuous to expect students to rely on their use only [4,5,9].

ORs offered unexpected and ambiguous results, as the performance of these students was not stable across questions, and their answers could reach both extremes of the score spectrum. ORs are intrinsically designed and thought of as an evolution of CPG, more accessible and applicable to practice. This aspect may emerge in the excellent quality of answers given by this group in the DW domain, where students were able to follow the detailed workup algorithms offered by OR, which can graphically synthesize even complex clinical scenarios. An interesting experience across teaching hospitals in Japan evidenced a somewhat significant positive correlation between the use of OR and score performance in a national general medicine test, in a numerous population of over 3000 residents. According to the authors, frequent logging into and consultation of UpToDate might have contributed to improved clinical reasoning skills, specifically in the tested domains of DD generation, and clinical decision deployment [16].

### Specific Observations Around the Students’ Use of Each Type of CDSS

#### CPG Group

For this group’s ability to reach a correct solution, the crucial step seemed to be selecting the proper guideline to consult. Once correctly identified, suitable CPG contain virtually everything a physician should know about a disease. During the initial process of elaboration of a clinical scenario, though, it is not immediately clear which disease entity, often more than one, is going to be selected as a candidate diagnosis for the patient. Choosing the right CPG can therefore be quite challenging. Additionally, CPG deliver their content in the form of plain text and interspersed summary tables and charts. The students had some difficulty in focusing on the right chart.

#### OR Group

Likely due to their lack of experience with the tool, the students failed to search for symptoms within the query. One hypothesis could be that they relied on their prior knowledge to make decisions rather than on the results obtained from each query, preventing them from breaking free from their preconceptions.

#### ChatGPT Group

Our results revealed students to lack substantial background on how to properly approach an LLM chatbot. *Masterprompting*, referred to as the assignment of the role and behavior expected from the chatbot, was not provided by the students.

Using an LLM as a CDSS inevitably introduces a “prompt bias,” for which the human subjective way of reasoning and choice of questions to be asked directly influence how the chatbot perceives the information and how it transforms its responses accordingly. Along such a general trend, the students’ prompts were not properly designed and translated into confused and misleading answers by ChatGPT. For example, clinical details were provided without any structure or further clinical context, triggering diverging suggestions by the chatbot. Accordingly, hints were given by the instructor on asking the LLM directly how to convey information for it to understand and elaborate on such information at its best, but consistent results did not follow.

On other instances, it was ChatGPT itself that derailed students’ reasoning. For example, a chatbot answer about laboratory values, lacking a relative unit of measurement and normal reference ranges, leads to misinterpretation of hypercalcemia as hypocalcemia, a critical mistake.

ChatGPT does not provide any literature citation and guideline reference to support its own line of reasoning, as other CDSS do in the form of easily accessible links to further explanations and deeper dives (eg differential diagnoses, varying DWs, tables of available first-line therapies, etc) [6]. Such an absence seemed to be associated with a significantly lower propensity of students to question either their own knowledge or the answers formulated by ChatGPT, blindly accepting the information provided. Whenever such indulgence was noted and pointed out by the instructors, students confessed how certain answers provided by ChatGPT were not completely clear and understandable; nonetheless, they willingly accepted them as valid.

### Limitations

Some limitations of this report must be underlined. First, the restricted number of students who took part in the experiment. This may have led to uneven distribution of differently ranked students in the groups, despite the random strategy used for group definition. Second, our methodological constraints lead to insufficient statistical power to draw sound conclusions. Lastly, the experiment has not yet been repeated, and the results have been further confirmed or discarded with other clinical cases.

### Conclusions

As in many other disciplines, the adoption of LLMs in medical practice and in the medical school curriculum is inevitable [10]. Our hands-on session suggests the critical need to include in medical degree courses teachings on how to properly take advantage of LLMs such as ChatGPT, as we verified that the potential for misuse is evident and real.

Our experience suggests the need for medical students to be acquainted with LLMs in their learning process and future profession. ChatGPT does not provide nor teach a reasoning method to approach a medical case resolution, a relevant issue for it to be recognized as part of the armamentarium in formal medical education. CPG and OR, on the contrary, most often provide step-by-step guidance on how to behave in each clinical scenario, how to approach diagnosis, and how to address treatment of diseases. References and recommendation strength form the cornerstone of these tools and help the student get progressively acquainted with ever-updating medical knowledge [4,5].

In conclusion, regarding the upcoming future, we suggest medical educators to:

- start to increasingly incorporate and refer to LLMs in their teachings, also by building tailored case studies [17-20];
- favor practice-based learning by using LLMs as a help to navigate guidelines and repositories with more ease and speed;
- exploit the very limitations of LLMs—such as the lack of an explicit reasoning method or unsure reliance on the latest published literature—to prompt students to consciously provide them themselves to the chatbot, turning the CDSS consultation process into a bidirectional teaching environment, possibly uncovering biases and misconceptions on both sides;
- help students in focusing on their accountability: they should be pushed to continuously look for evidence and validation of their own clinical reasoning, avoiding relying completely on that of LLMs.

## Supplementary material

10.2196/55709 Multimedia Appendix 1 ChatGPT conversation history for clinical case generation.

10.2196/55709 Multimedia Appendix 2 Clinical Case and questions for students.

10.2196/55709 Multimedia Appendix 3 Workflow Diagram.

## Abbreviations

CDM: clinical decision-making
CDSS: clinical decision support systems
CPG: clinical practice guidelines
DD: differential diagnosis
DW: diagnostic workup
LLM: large language model
OR: online repositories
