# Supplementary material for: Impact of Clinical Decision Support Systems on Medical Students’ Case-Solving Performance: Comparison Study with a Focus Group
Source: JMIR Med Educ. 2025 Mar 18;11:e55709. doi: 10.2196/55709 (PMC11936302; doi:10.2196/55709)
Supplement: Multimedia Appendix 1 [file mededu-v11-e55709-s001.docx]

**You**

We want you to invent a fictional clinical case about a 72 years old woman with antiphospholipid syndrome diagnosed due to multiple abortions but no major thrombotic events presenting to the ER because of abdominal pain and muscle weakness. Specify the medication history of the patient before this event.

It comes out that she has hypercalcemia because of osteolytic metastases due to an undiagnosed breast cancer. She is admitted to the general medicine ward where she develops hospital acquired pneumonia requiring specific antibiotics and hydrocortisone. The infective insult and ion disturbance also lead to atrial fibrillation, which is cardioverted.

During the hospital stay a breast biopsy is obtained and a ER+ adenocarcinoma is diagnosed. State the therapy that is proposed to the patient accordingly.

Provide details about recorded vitals, performed lab exams, radiological reports, invasive procedures and administered therapies.

**ChatGPT**

Clinical Case:

Patient Profile:

Name: Mrs. Elizabeth Thompson

Age: 72 years old

Gender: Female

Medical History:

Antiphospholipid syndrome: Diagnosed due to multiple abortions.

No major thrombotic events reported.

Medication History:

Aspirin: Prescribed for antiphospholipid syndrome.

Folic acid: Prescribed for its potential benefits in antiphospholipid syndrome.

Presenting Complaint:

Abdominal pain and muscle weakness.

Initial Assessment:

Mrs. Thompson presented to the Emergency Room complaining of abdominal pain and muscle weakness.

Upon physical examination, she appeared weak and fatigued.

Recorded vitals:

Blood pressure: 140/90 mmHg

Heart rate: 92 bpm

Respiratory rate: 18 breaths per minute

Temperature: 37.2°C (oral)

Laboratory Exams:

Complete blood count:

Hemoglobin: 11.2 g/dL (slightly below normal range)

Platelet count: 205,000/mm³ (within normal range)

White blood cell count: 8,600/mm³ (within normal range)

Coagulation profile:

Prothrombin time (PT): 12.5 seconds (within normal range)

Activated partial thromboplastin time (aPTT): 32 seconds (within normal range)

Renal function:

Blood urea nitrogen (BUN): 24 mg/dL (slightly elevated)

Creatinine: 1.2 mg/dL (within normal range)

Liver function:

Alanine aminotransferase (ALT): 32 U/L (within normal range)

Aspartate aminotransferase (AST): 28 U/L (within normal range)

Serum calcium level: 11.2 mg/dL (elevated)

Radiological Reports:

Abdominal X-ray: No significant abnormalities detected.

Chest X-ray: Normal findings.

Invasive Procedures:

Breast biopsy: Due to the discovery of osteolytic metastases, a breast biopsy is performed to investigate the underlying cause. The biopsy results reveal an ER+ adenocarcinoma.

Diagnosis:

Mrs. Thompson is diagnosed with hypercalcemia caused by osteolytic metastases resulting from an undiagnosed breast cancer.

Therapy Proposed:

Treatment for Mrs. Thompson's ER+ adenocarcinoma includes a multidisciplinary approach involving oncology, surgery, and radiation therapy.

Proposed therapy:

Surgery: Lumpectomy or mastectomy to remove the breast tumor and nearby lymph nodes.

Radiation therapy: External beam radiation to target any remaining cancer cells in the breast area.

Hormonal therapy: Administration of antiestrogen drugs (such as tamoxifen or aromatase inhibitors) to block the effects of estrogen and inhibit tumor growth.

Chemotherapy: Depending on the cancer stage, chemotherapy may be recommended to target potential metastases or to reduce the risk of recurrence.

Additional Interventions:

Hospital-acquired pneumonia: Mrs. Thompson develops hospital-acquired pneumonia during her stay.

Specific antibiotics: A course of broad-spectrum antibiotics, such as levofloxacin or ceftriaxone, is initiated to treat the pneumonia.

Hydrocortisone: To manage the patient's hypercalcemia and its effects, hydrocortisone is administered as it can help reduce bone resorption.

Cardioversion for Atrial Fibrillation:

Mrs. Thompson develops atrial fibrillation as a result of the infective insult and electrolyte disturbances.

Cardioversion: The atrial fibrillation is cardioverted using electrical shocks to restore a normal heart rhythm.

Overall, Mrs. Thompson's case involves managing the antiphospholipid syndrome, hypercalcemia due to osteolytic metastases, ER+ adenocarcinoma of the breast, hospital-acquired pneumonia, and atrial fibrillation. The treatment plan includes surgery, radiation therapy, hormonal therapy, and potential chemotherapy for the breast cancer, along with antibiotics and hydrocortisone for the pneumonia and hypercalcemia.

**You**

how would you treat a severe hypercalcemia?

**ChatGPT**

Severe hypercalcemia requires prompt treatment to prevent complications and restore normal calcium levels. The specific treatment approach may vary depending on the underlying cause of hypercalcemia. Here are some general measures and interventions commonly employed in the management of severe hypercalcemia:

Fluid Replacement: The first step is usually aggressive hydration with intravenous fluids to promote urinary calcium excretion and enhance renal function. Normal saline (0.9% sodium chloride) is commonly used for this purpose.

Loop Diuretics: In cases where there is adequate urine output, loop diuretics like furosemide may be administered to further enhance calcium excretion.

Bisphosphonates: Bisphosphonates, such as zoledronic acid or pamidronate, are commonly used medications to rapidly lower calcium levels. They work by inhibiting bone resorption and reducing calcium release from the bones. These medications are usually administered intravenously over several hours or days.

Calcitonin: Calcitonin is a hormone that inhibits bone resorption and enhances urinary calcium excretion. It can be administered intravenously or intramuscularly for short-term management of severe hypercalcemia.

Glucocorticoids: In some cases, glucocorticoids like prednisone may be used to reduce calcium levels, especially if hypercalcemia is due to increased production of calcitriol (active form of vitamin D) or granulomatous diseases.

Hemodialysis: In refractory cases or when other treatment options are contraindicated, hemodialysis may be considered. It is an effective method to rapidly reduce calcium levels by directly removing calcium from the blood.

Treatment of Underlying Cause: It is essential to identify and treat the underlying cause of hypercalcemia. In the case of osteolytic metastases, management of the primary malignancy, such as surgery, radiation therapy, or chemotherapy, is crucial.

It is important to note that the specific treatment plan for severe hypercalcemia should be tailored to the individual patient's condition and guided by the expertise of the healthcare team. Close monitoring of calcium levels, electrolytes, renal function, and hydration status is essential throughout the treatment process.

**You**

which would be the PTH, vitamin D, TSH, albumin, serum electrophoresis of this patient? invent

**ChatGPT**

Laboratory Results:

Parathyroid Hormone (PTH):

PTH: 35 pg/mL (Normal range: 10-65 pg/mL)

Vitamin D:

25-hydroxyvitamin D: 25 ng/mL (Normal range: 20-50 ng/mL)

Thyroid-Stimulating Hormone (TSH):

TSH: 1.8 mIU/L (Normal range: 0.4-4.0 mIU/L)

Albumin:

Albumin: 4.2 g/dL (Normal range: 3.5-5.0 g/dL)

Serum Electrophoresis:

Protein electrophoresis shows normal levels and distribution of serum proteins.

Please note that these values are fictional and do not reflect actual patient data. The interpretation of laboratory results should be done in conjunction with clinical findings and the patient's specific medical history.

**You**

Which exams would you do for the workup of low-pth hypercalcemia?

**ChatGPT**

When investigating low-PTH (parathyroid hormone) hypercalcemia, it is essential to evaluate the underlying cause. Several diagnostic tests may be conducted to aid in the workup of this condition. Here are some commonly performed exams and investigations:

Serum Calcium Level: Measure the serum calcium concentration to confirm hypercalcemia and assess the severity.

PTH Level: Although low PTH is already established, measuring the PTH level can help confirm the primary cause of hypercalcemia and rule out other potential disorders.

25-Hydroxyvitamin D Level: Evaluate the levels of 25-hydroxyvitamin D to assess vitamin D status. Low vitamin D levels can contribute to hypercalcemia.

1,25-Dihydroxyvitamin D Level: Measure the active form of vitamin D to assess its role in calcium regulation. Elevated levels may indicate excess vitamin D production or intake.

Renal Function Tests: Perform blood tests such as blood urea nitrogen (BUN), creatinine, and estimated glomerular filtration rate (eGFR) to evaluate kidney function. Hypercalcemia can affect renal function, and underlying kidney disease may contribute to calcium dysregulation.

Ionized Calcium: Measure the concentration of ionized calcium, as it represents the physiologically active form and can provide more accurate information about calcium status.

Complete Blood Count (CBC): Assess for any signs of underlying hematologic abnormalities or malignancies that may be causing hypercalcemia.

Imaging Studies:

Chest X-ray: Conduct a chest X-ray to evaluate for potential lung pathologies, such as granulomatous diseases.

Abdominal Ultrasound/CT Scan: Evaluate the abdomen for the presence of malignancies, including liver, kidney, or adrenal tumors, which can cause hypercalcemia.

Bone Mineral Density (BMD) Test: Measure bone density using dual-energy X-ray absorptiometry (DEXA scan) to assess for potential osteoporosis or bone disorders.

Genetic Testing: In some cases, genetic testing may be considered, especially if hereditary causes of hypercalcemia, such as familial hypocalciuric hypercalcemia (FHH), are suspected.

The specific tests ordered will depend on the individual patient's clinical presentation, medical history, and initial laboratory findings. It is important to consult with a healthcare professional for proper evaluation and interpretation of test results.

**You**

invent a PTH related protein value for this patient

**ChatGPT**

Laboratory Result:

PTH-Related Protein (PTHrP) Level:

PTHrP: 2.5 pmol/L (Normal range: 0-2.0 pmol/L)

Please note that this value is fictional and does not reflect actual patient data. PTHrP is a protein that can mimic the actions of PTH and cause hypercalcemia. Elevated levels of PTHrP can be seen in certain malignancies, such as squamous cell carcinoma of the lung, breast, or head and neck. Measurement of PTHrP helps assess its potential contribution to hypercalcemia and guide further diagnostic investigations. Interpretation of laboratory results should be done in conjunction with clinical findings and the patient's specific medical history.

**You**

invent the bone scintigraphy report

**ChatGPT**

Bone Scintigraphy Report:

Indication: Evaluation of bone metastases in a patient with known breast adenocarcinoma and hypercalcemia.

Findings:

Whole-body bone scintigraphy was performed using technetium-99m methylene diphosphonate (99mTc-MDP) radiotracer.

There are multiple areas of increased radiotracer uptake noted throughout the skeletal system, consistent with widespread bone metastases.

The most significant areas of abnormal tracer uptake are observed in the spine, pelvis, ribs, and bilateral femurs.

The metastatic lesions appear as areas of focal intense uptake, demonstrating an osteoblastic pattern.

The scintigraphic findings are indicative of diffuse skeletal involvement, suggesting advanced metastatic disease.

Impression:

Diffuse skeletal metastases noted, consistent with widespread osseous involvement secondary to breast adenocarcinoma.

Please note that this report is fictional and does not reflect actual patient data. Bone scintigraphy is a nuclear medicine imaging technique commonly used to detect bone metastases. The scintigraphy findings described above indicate extensive metastatic involvement of the skeletal system in the context of breast adenocarcinoma. Proper interpretation and correlation of imaging findings should be done by a qualified healthcare professional.

**You**

invent a mammography report

**ChatGPT**

Mammography Report:

Patient Information:

Name: Mrs. Elizabeth Thompson

Age: 72 years

Gender: Female

Clinical Presentation:

Diagnosis of ER+ adenocarcinoma of the breast.

Mammography Findings:

Bilateral digital mammography was performed using standard projections and techniques.

Right Breast: The mammogram reveals a 2.5 cm irregular mass with speculated margins in the upper outer quadrant of the right breast. The mass demonstrates high density and microcalcifications.

Left Breast: No suspicious masses or significant architectural distortions are identified. No calcifications or other concerning findings are observed.

Impression:

Suspicious irregular mass with speculated margins and microcalcifications in the upper outer quadrant of the right breast, highly suggestive of malignancy.

No significant abnormalities detected in the left breast.

Recommendation:

Based on the mammography findings, further evaluation with a breast biopsy is strongly advised to establish a definitive diagnosis and determine the extent of malignancy.

Clinical correlation and consultation with a breast surgeon or oncologist for treatment planning are recommended.

Please note that this report is fictional and does not reflect actual patient data. Mammography is a commonly used imaging modality for breast cancer screening and diagnosis. The findings described above are indicative of suspicious findings in the right breast, raising concerns for malignancy. However, the interpretation and final diagnosis should be performed by a qualified radiologist after careful assessment of the mammogram.

**You**

Invent a physical examination at the admission of this patient

**ChatGPT**

Physical Examination at Admission:

General Appearance:

Mrs. Elizabeth Thompson appears weak and fatigued.

She is alert and oriented to person, place, and time.

Vital signs are stable, as mentioned previously.

Head and Neck:

No palpable lymphadenopathy in the cervical, supraclavicular, or axillary regions.

Inspection of the head and neck reveals no visible masses or abnormalities.

No jugular venous distention is noted.

Chest and Lungs:

Symmetrical chest wall expansion.

Auscultation of the lungs reveals normal vesicular breath sounds bilaterally.

No adventitious sounds such as crackles, wheezes, or decreased breath sounds are heard.

Cardiovascular System:

Regular heart rhythm on auscultation.

S1 and S2 heart sounds are normal, with no murmurs, rubs, or gallops appreciated.

Peripheral pulses are palpable and symmetrical.

Abdomen:

Mild tenderness to palpation in the lower abdomen, predominantly in the right upper quadrant.

No palpable masses or organomegaly.

Bowel sounds are present in all quadrants.

Musculoskeletal System:

Muscle strength assessment reveals generalized weakness.

No joint swelling, deformities, or limited range of motion noted.

Neurological Examination:

Cranial nerves: Intact, no abnormalities observed.

Motor function: Decreased muscle strength noted globally.

Sensory function: No focal sensory deficits detected.

Reflexes: Deep tendon reflexes are diminished but present symmetrically.

Skin:

No rashes, lesions, or skin abnormalities observed.

No signs of hyperpigmentation or jaundice.

Please note that this physical examination is fictional and does not reflect actual patient data. A comprehensive physical examination is an essential part of the clinical assessment and may vary depending on the patient's presentation and specific findings. The examination described above provides a general overview of the assessment at the time of admission for Mrs. Thompson. A complete evaluation should be conducted by a healthcare professional based on the patient's individual case.

**You**

rewrite with some confounding findings

**ChatGPT**

Physical Examination at Admission:

General Appearance:

Mrs. Elizabeth Thompson appears weak and fatigued.

She is alert and oriented to person, place, and time.

Vital signs are stable, except for an elevated heart rate of 110 beats per minute.

Head and Neck:

No palpable lymphadenopathy in the cervical, supraclavicular, or axillary regions.

Inspection of the head and neck reveals pallor and dry mucous membranes.

No jugular venous distention is noted.

Chest and Lungs:

Symmetrical chest wall expansion.

Auscultation of the lungs reveals decreased breath sounds at the bases bilaterally.

Fine crackles are heard in the lower lung fields.

Cardiovascular System:

Irregular heart rhythm on auscultation.

S1 and S2 heart sounds are normal, with no murmurs appreciated.

Peripheral pulses are weak and thready.

Abdomen:

Mild tenderness to palpation in the lower abdomen, predominantly in the right upper quadrant.

No palpable masses or organomegaly.

Bowel sounds are diminished.

Musculoskeletal System:

Muscle strength assessment reveals generalized weakness.

Mild swelling and tenderness noted in the joints of the hands and knees.

Limited range of motion observed in the affected joints.

Neurological Examination:

Cranial nerves: Intact, no abnormalities observed.

Motor function: Decreased muscle strength noted globally.

Sensory function: No focal sensory deficits detected.

Reflexes: Deep tendon reflexes are diminished but present symmetrically.

Skin:

Pale skin with cool extremities.

No rashes or lesions observed.

No signs of hyperpigmentation or jaundice.

Please note that this revised physical examination includes confounding findings to reflect a more complex clinical presentation. These fictional findings are meant for illustrative purposes only and do not represent actual patient data. A thorough evaluation should be conducted by a healthcare professional to assess the patient's condition accurately and guide appropriate management.

**You**

rewrite considering the same person but at 52 years old

**ChatGPT**

Physical Examination at Admission:

General Appearance:

Mrs. Elizabeth Thompson appears tired but well-groomed.

She is alert and oriented to person, place, and time.

Vital signs are within normal limits for her age.

Head and Neck:

No palpable lymphadenopathy in the cervical, supraclavicular, or axillary regions.

Inspection of the head and neck reveals no visible masses or abnormalities.

No jugular venous distention is noted.

Chest and Lungs:

Symmetrical chest wall expansion.

Auscultation of the lungs reveals clear breath sounds bilaterally.

No adventitious sounds such as crackles, wheezes, or decreased breath sounds are heard.

Cardiovascular System:

Regular heart rhythm on auscultation.

S1 and S2 heart sounds are normal, with no murmurs, rubs, or gallops appreciated.

Peripheral pulses are palpable and symmetrical.

Abdomen:

No tenderness to palpation in the abdomen.

No palpable masses or organomegaly.

Bowel sounds are present in all quadrants.

Musculoskeletal System:

Muscle strength assessment reveals normal strength and tone.

No joint swelling, deformities, or limited range of motion noted.

Neurological Examination:

Cranial nerves: Intact, no abnormalities observed.

Motor function: Normal muscle strength and coordination.

Sensory function: No focal sensory deficits detected.

Reflexes: Deep tendon reflexes are present and symmetrical.

Skin:

No rashes, lesions, or skin abnormalities observed.

Normal skin color and moisture.

Please note that this physical examination is fictional and does not reflect actual patient data. It reflects a physical examination of Mrs. Thompson at the age of 52, without the presence of significant abnormalities. A comprehensive evaluation should be conducted by a healthcare professional based on the patient's specific presentation and individual case.

**You**

Rewrite with confounding findings

**ChatGPT**

Physical Examination at Admission:

General Appearance:

Mrs. Elizabeth Thompson appears fatigued and visibly distressed.

She is alert but appears anxious.

Vital signs show an elevated heart rate of 110 beats per minute and elevated blood pressure.

Head and Neck:

No palpable lymphadenopathy in the cervical, supraclavicular, or axillary regions.

Inspection of the head and neck reveals dry skin and cracked lips.

No jugular venous distention is noted.

Chest and Lungs:

Symmetrical chest wall expansion.

Auscultation of the lungs reveals decreased breath sounds with scattered crackles throughout both lung fields.

Wheezing is heard upon expiration.

Cardiovascular System:

Irregular heart rhythm on auscultation.

S1 and S2 heart sounds are normal, with an audible systolic murmur.

Peripheral pulses are weak and thready.

Abdomen:

Mild tenderness to palpation in the epigastric region.

No palpable masses or organomegaly.

Bowel sounds are hyperactive.

Musculoskeletal System:

Muscle strength assessment reveals generalized weakness.

Joint swelling and tenderness observed in multiple joints, including hands and knees.

Limited range of motion in affected joints.

Neurological Examination:

Cranial nerves: Intact, but the patient appears restless.

Motor function: Decreased muscle strength noted globally.

Sensory function: No focal sensory deficits detected.

Reflexes: Deep tendon reflexes are diminished but present symmetrically.

Skin:

Pale and cool skin with delayed capillary refill.

No rashes or lesions observed.

No signs of hyperpigmentation or jaundice.

Please note that this revised physical examination includes confounding findings to reflect a more complex clinical presentation. These fictional findings are meant for illustrative purposes only and do not represent actual patient data. A thorough evaluation should be conducted by a healthcare professional to assess the patient's condition accurately and guide appropriate management.
