# Supplementary material for: Impact of Clinical Decision Support Systems on Medical Students’ Case-Solving Performance: Comparison Study with a Focus Group
Source: JMIR Med Educ. 2025 Mar 18;11:e55709. doi: 10.2196/55709 (PMC11936302; doi:10.2196/55709)
Supplement: Multimedia Appendix 2 [file mededu-v11-e55709-s002.pdf]

Clinical case presentation

54-year-old woman

Social History
Married, one children, did not breastfeed. School teacher. Current smoker. Drinks 1 UA per week.

Family History
Her mother had type 2 diabetes, and her father had hypertension. No significant family history of kidney disease or heart failure.

Past Medical History
- **Arterial hypertension**
- **Antiphospholipid Syndrome** diagnosed due to multiple abortions. No major thrombotic events reported.

Medications:
- valsartan + hydrochlorothiazide 160/12.5 mg qd
- aspirin 100 mg qd

History of presenting illness
Referred to your internal medicine clinics for **worsening abdominal pain** and **progressive muscle weakness** in the past 3 weeks

Vital signs
- Blood pressure: 140/90 mmHg
- Heart rate: 110 bpm
- Respiratory rate: 18 breaths per minute
- Oxygen saturation: 97% on room air
- Temperature: 37.2° C (oral)

Physical Examination
- Appears well groomed. Weak and fatigued, yet anxious. She is alert and oriented to person, place, and time.
- *Head and Neck*: No palpable lymphadenopathy in the cervical or supraclavicular. Inspection of the head and neck reveals pallor and dry mucous membranes. No jugular venous distention is noted.
- *Chest and Lungs*: Auscultation of the lungs reveals decreased breath sounds at the bases bilaterally. Fine crackles are heard in the lower lung fields.
- *Cardiovascular System*: Regular heart rhythm on auscultation. Systolic murmur 2/6 noted on mitral focus.
- *Abdomen*: Mild tenderness to palpation in the lower abdomen, predominantly in the right upper quadrant. No palpable masses or organomegaly. Bowel sounds are diminished.
- *Musculoskeletal System*: Muscle strength assessment reveals generalized weakness. Mild swelling and tenderness noted in the joints of the hands and knees. Normal range of motion observed.
- *Neurological Examination*: Cranial nerves: Intact, no abnormalities observed. Motor function: Decreased muscle strength noted globally. Sensory function: No focal sensory deficits detected.
- *Reflexes*: Deep tendon reflexes are diminished but present symmetrically.
- *Skin*: Pale skin with cool extremities. Light diffuse erythematous rash on dorsum, vanishing on finger pressure. No signs of jaundice.

Question 1

You are the attending physician:

Rank the possible differential diagnoses in terms of probability.

Question 2

Based on the previous list: **which diagnostic workup would you set up?**

Initial laboratory values at hospital admission

- **CBC**: WBC 8400/uL (N 2000), Hb 11.2 g/dL (MCV 78 fL), PLT 300000/uL
- **Kidney & electrolytes**: Creatinine 2,5 mg/dL, Urea 50 mg/dL, Na 132 mmol/L, K 3.3 mmol/L, Ca 4 mmol/L
- **Muscle**: CPK 98 U/L
- **Liver**: AST 53 U/L, ALT 64 U/L, GGT 71 U/L, Bili-tot 0,7 mg/dL
- **Coagulation**: PT 13.8 seconds, aPTT: 35 seconds, INR: 1.2
- **Autoimmunity**: myositis panel neg, ANA pos, anti-SCL70 and anti-centromere neg
- **Systemic inflammation**: CRP 22 mg/L, ESR 35 mm/hr

Question 3

Which values are **altered?**

Question 4

We are in front of a case of severe hypercalcemia and AKI.
Which treatment do you start?

Question 5

Which are the **possible causes of hypercalcemia?**

Additional data

Endocrinological panel

- PTH: 5 pg/mL
- 25-hydroxyvitamin D: 23 ng/mL
- TSH: 1.8 mIU/L

Albumin: 4.2 g/dL
Protein electrophoresis shows normal levels and distribution of serum proteins.

Question 6

Can you **narrow down the previous list** based on these findings?

Based on the age of the patient and blood exam results...

...a possible diagnosis is **hypercalcemia of malignancy**.

Question 7

Which are the **primary diagnostic tests** that you order?

Diagnostic workup performed:

PTHrp: **2.5 pmol/L** (Normal range: 0-2.0 pmol/L)

Mammography: reveals a 2.5 cm irregular mass with speculated margins in the upper outer quadrant of the right breast. The mass demonstrates high density and microcalcifications.
Bone scintigraphy: significant areas of abnormal tracer uptake are observed in the pelvis, ribs, and bilateral femurs
Full-body CT scan: presence multiple osteolytic lesions (VII-VIII right costae, left femur); clear lung parenchyma; intact abdominal organs

--> Breast nodule biopsy: **Er+/Her2- breast adenocarcinoma**

Question 8

Which **therapeutic choice** do you offer the patient?
