# Supplementary figures and images for: Impact of Clinical Decision Support Systems on Medical Students’ Case-Solving Performance: Comparison Study with a Focus Group
Source: JMIR Med Educ. 2025 Mar 18;11:e55709. doi: 10.2196/55709 (PMC11936302; doi:10.2196/55709)

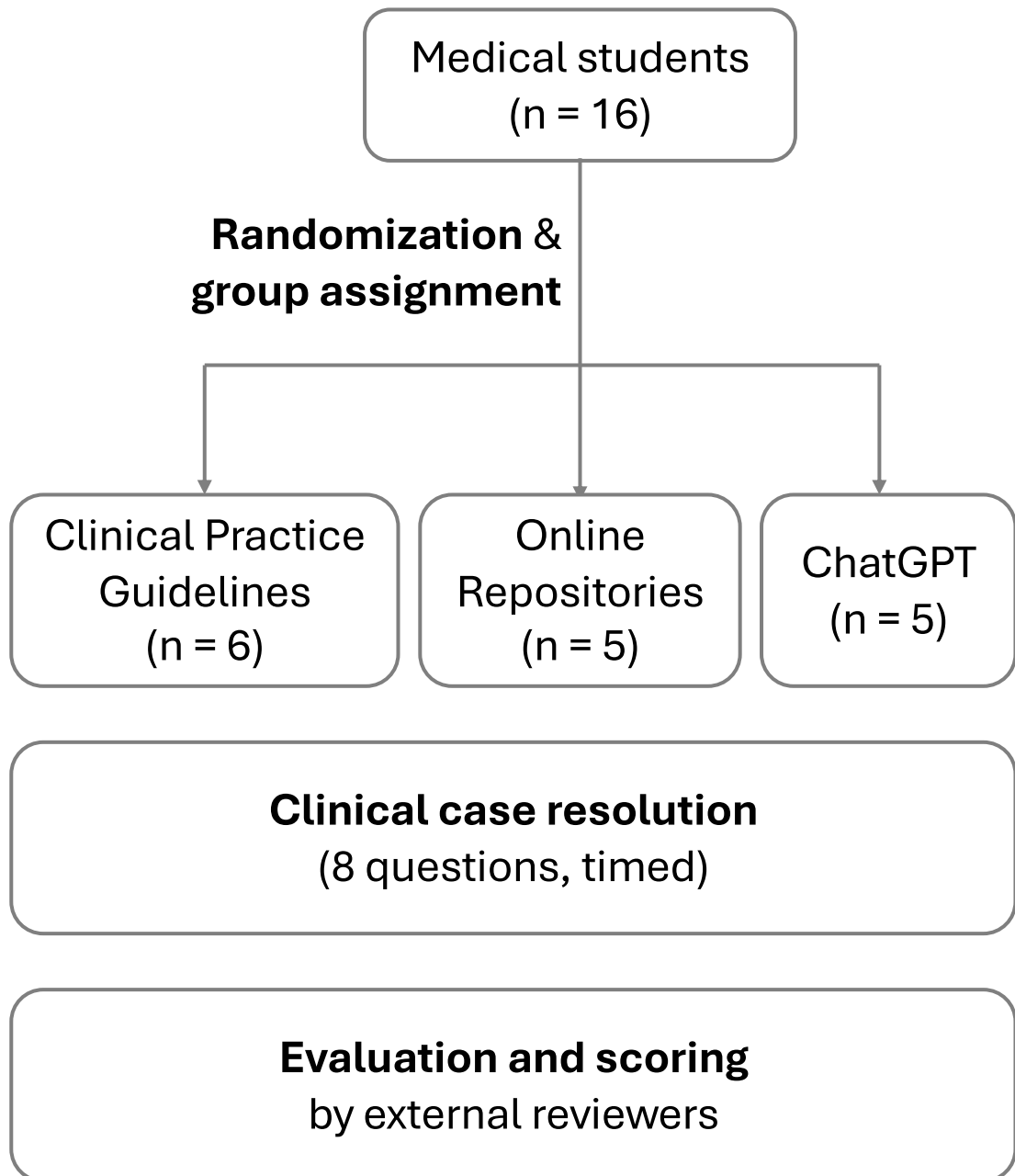

Supplement: Multimedia Appendix 3 [file mededu-v11-e55709-s003.pdf]
